# Semi-Rigid Fixation Using a Sliding Plate for Treating Fractures of the Mandibular Condylar Process

**DOI:** 10.3390/jcm10245782

**Published:** 2021-12-10

**Authors:** Byung-Kyu So, Kyeong-Soo Ko, Dong-Hyuck Kim, Hyon-Seok Jang, Eui-Seok Lee, Ho-Kyung Lim

**Affiliations:** 1 Department of Oral and Maxillofacial Surgery, Korea University Guro Hospital, Seoul 08308, Korea; sobk4728@naver.com (B.-K.S.); k1015ks@naver.com (K.-S.K.); hyperlion@naver.com (D.-H.K.); 2 Department of Oral and Maxillofacial Surgery, Korea University Ansan Hospital, Ansan 15355, Korea; omfs1109@korea.ac.kr

**Keywords:** semi-rigid fixation, mandibular condyle fracture, siding plate

## Abstract

Occlusal displacement often occurs after surgery for condylar process fractures because it is difficult to reduce these fractures precisely. However, performing semi-rigid fixation using a sliding plate may overcome this limitation. A retrospective clinical comparison between semi-rigid and rigid fixations was performed. Among 34 patients who had unilateral condylar process fractures, 17 were treated with rigid fixation and the remaining with semi-rigid fixation using a sliding plate. For all patients, panoramic radiographs were collected 1 day and 6 months after surgery. In these radiographs, ramus height and condylar process inclination were measured, and the differences between the fractured and normal sides were assessed. Additionally, the radiographic density of the fracture area was measured. Differences in surgical outcomes and operative times between the two groups and changes in postoperative deviations within each group were analyzed. There was no statistically significant difference in ramus height and condylar process inclination between the two groups at postoperative day 1 and 6 months. Radio-density was observed to be higher in the rigid fixation group, and it increased with time in both groups. The semi-rigid fixation group had a significantly shorter operative time than the other group did. Semi-rigid and rigid fixations showed no differences in terms of effectiveness and outcomes of surgery. In terms of operative time, semi-rigid fixation was superior to rigid fixation.

## 1. Introduction

Condylar process fractures of the mandible account for a large percentage of maxillofacial trauma. Most cases are treated surgically, except for intracapsular fractures in minimally displaced cases [1,2]. Many procedures and devices have been used for the internal fixation of condylar process fractures. Wires and lag screws have been popularly used in the past, and plate and screw systems are used by most surgeons nowadays. The plate system is easier to position than lag screws, is more stable than wires, and has the suitable tensile strength to maintain fixation [3]. However, rigid fixation may not be achieved if the plate is not correctly located along the strain-stress line, and multiple plates may be required for rigid fixation; additionally, it may be difficult to apply plates to the desired location if the intraoral approach is used, or the condylar segment is small [4]. Moreover, if internal fixation is finished without complete reduction, displacement of the condyle may result in not only interference with mandibular movement but also the need for additional surgery.

In this study, we propose the use of sliding plates for the open reduction of mandibular condylar process fractures. In fact, in sagittal split ramus osteotomy, fixation between segments using sliding plates has been considered in some studies, and improved convenience and sufficient stability have been reported [5,6]. In cases of condylar process fractures, the aim is to prevent lateral displacement of the condylar segment and correct surgical error through the function of the masticatory neuromuscular system; therefore, more precise occlusion could be achieved by using sliding plates in open reduction. In this study, we compared semi-rigid fixation using sliding plates with traditional rigid fixation using a traditional four-hole miniplate in terms of stability, convenience, and prognosis.

## 2. Materials and Methods

### 2.1. Study Design and Patient Selection Criteria

A retrospective case-control study was conducted. A total of 34 patients who underwent open reduction via an intraoral or extraoral approach for a unilateral condylar fracture of the mandible from 2007 to 2015 at the Department of Oral and Maxillofacial Surgery, Korea University Guro Hospital were included in this study. Patients under 18 years of age, with severe facial asymmetry, bilateral condylar fracture, condylar head fracture, crushed condylar fracture, a completely edentulous state, and systemic compromised conditions of Class III or more per ASA (American Society of Anesthesiologists) classification were excluded from the study. Patients were classified into two groups (*n* = 17) according to the plates used during the surgery as follows: those who underwent fixation using sliding plates were classified as a semi-rigid fixation group and those who underwent fixation using a traditional four-hole miniplate as a rigid fixation group. The study was conducted according to the guidelines of the Declaration of Helsinki and approved by the Institutional Review Board of Korea University (no. 2015GR0714).

### 2.2. Surgical Procedures

**Figure 1:**
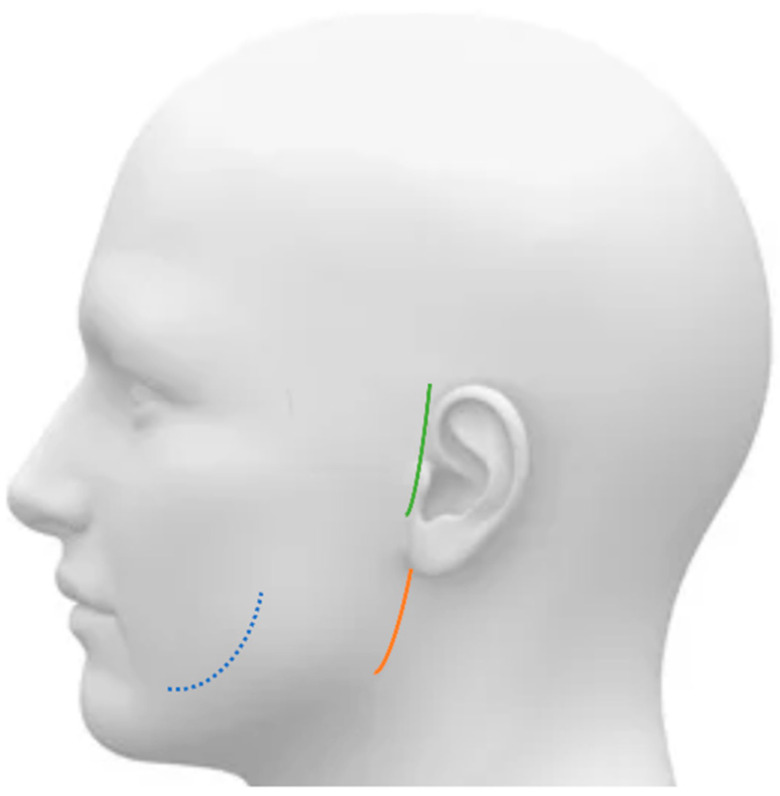
The 3 different approaches—intraoral (blue dash), retromandibular (orange line), or preauricular (green line)—for open reduction of the mandibular condylar process.

The baseline of the approach and fixation protocols used during the surgery were based on AOCMF (Arbeitsgemeinschaft für Osteosynthesefragen Craniomaxillofacial) surgery reference [7]. In this study, the intraoral, retromandibular, or preauricular approach was used for open reduction of the mandibular condylar process along with the level of the fracture line (Figure 1). In most cases of subcondyle fracture, the intraoral approach was adopted; in most cases of the neck fracture, the retromandibular approach was adopted; and in most cases of head fracture, the preauricular approach was adopted.

In the intraoral approach, local anesthesia was achieved with lidocaine containing 1:100,000 epinephrine (Huons, Seongnam, Gyeonggi-do, Korea) in the mandibular posterior buccal vestibule area. The incision was made from the first molar to the external oblique ridge at a distance of 5 mm from the mucogingival junction. Subperiosteal dissection was performed to expose the fracture line sufficiently with a periosteal elevator. For internal fixation, an additional approach with a stab incision was made on the buccal skin, and drilling and fixation were made using a trocar.

In the retromandibular approach, after local anesthesia was administered on the skin, a vertical incision was made on the skin and subcutaneous tissue from just below the ear lobe to the mandibular angle area along the posterior border of the mandible at a distance of 0.5 cm. After the subcutaneous tissue was dissected, and the superficial musculoaponeurotic system was exposed, a vertical incision was made on the parotid gland tissue. After a facial nerve branch was detected, blunt dissection was performed parallel to the facial nerve to reach the posterior border of the mandible. Next, the pterygomandibular sling was incised, and dissection of the masseter muscle and exposure of the fracture line was performed.

In the preauricular approach, after local anesthesia was administered on the skin, an incision was made on the skin and subcutaneous tissue along the preauricular skin crease. After the subcutaneous tissue was dissected to detect the temporalis fascia, the superficial layer of the temporalis fascia was incised to expose the surface of the zygomatic arch. Thereafter, an additional dissection was performed to expose the temporomandibular joint (TMJ) capsule and fracture line.

**Figure 2:**
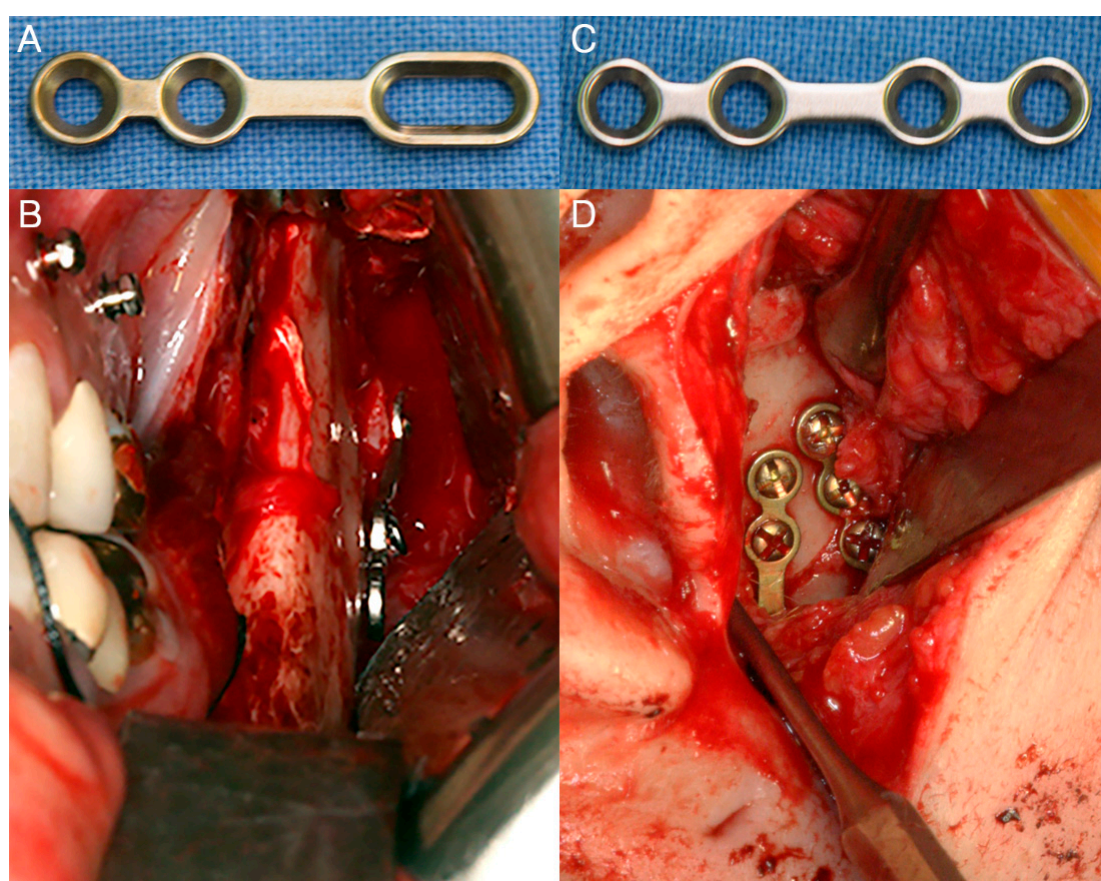
(**A**) Sliding plate (Biomaterials Korea Inc., Hanam, Gyeonggi-do, Korea); (**B**) sliding plate applied on the mandibular condyle; (**C**) four-hole miniplate (Stryker Corp., Kalamazoo, MI, USA); (**D**) four-hole miniplate applied on the mandibular condyle.

After reduction of the fractured segment was performed in the right position, internal fixation was conducted after intermaxillary fixation (IMF) using an Erich arch bar or a screw-assisted system (Stryker Corp., Kalamazoo, MI, USA). One sliding plate (Biomaterials Korea Inc., Hanam, Gyeonggi-do, Korea) (1.5-mm thickness) was used for each patient in the semi-rigid fixation group (Figure 2A). An oval-shaped screw hole was placed on the condylar segment to allow movement, and as much as possible, the plate was placed on the posterior border and fixed parallel to the central axis of the condylar process. The screw placed in the oval-shaped screw hole was fully tightened and then loosened approximately half a turn such that the condylar segment was free to move within the space of the hole [6] (Figure 2B). Two four-hole plates (Stryker Corp., Kalamazoo, MI, USA) (1.5-mm thickness) were used in each patient in the rigid fixation group; according to known compression and traction lines in the mandibular ramus, one was placed at the anterior border of the condylar process and the other at the posterior border of the condylar process [8] (Figure 2C,D). After the internal fixation was completed, an interrupted suture was performed with 4/0 Vicryl (Johnson & Johnson, New Brunswick, NJ, USA) in the mucosal incision area. For the extraoral approach, suturing was performed with 4/0 Vicryl at the muscular and subcutaneous levels and with 5/0 nylon (Johnson & Johnson, New Brunswick, NJ, USA) at the skin level.

Postoperative antibiotics (Cefminox; Meicelin, Yungjin Pharm, Songpa-Gu, Seoul, Korea) and non-steroidal anti-inflammatory drugs were administered for 7 days. For the patients in the semi-rigid fixation group, tight IMF with wiring for 2 weeks was maintained, followed by active mouth-opening practice. For the patients in the rigid fixation group, loose IMF with elastic rings for 2 weeks was maintained, and active mouth-opening practice was then performed.

### 2.3. Clinical and Radiological Evaluation

Clinical information, including age, sex, approach technique, and fracture site, was collected from patients’ medical records. The fracture site of the condylar process was classified as head, neck, and base of the condylar process according to the AOCMF criteria [9]. Complications, including mouth-opening limitation, early occlusal contact interference, facial nerve damage, acute infection, condylar head resorption, and malunion, were also recorded. In addition, the presence or absence of malocclusion and TMJ disorders were investigated. The operative time of both procedures was investigated through anesthesia records and compared to analyze the effect of fixation methods on operative time.

**Figure 3:**
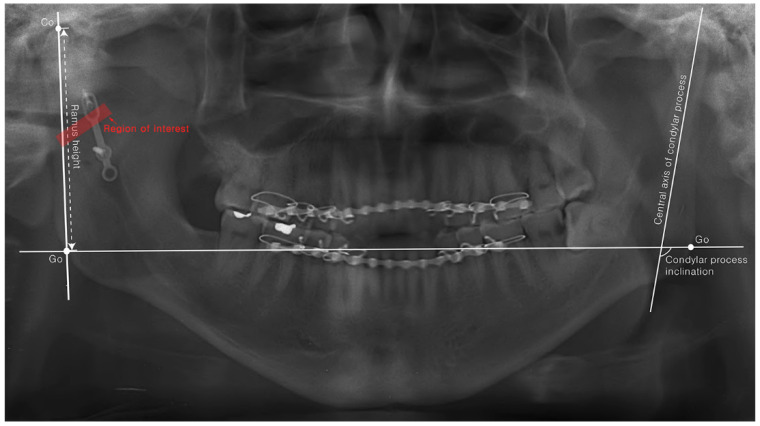
Measurement guidelines for ramus height, condylar process inclination, and radiodensity. Ramus height was defined as the distance from the point of the mandibular angle (gonion, Go) to the uppermost point of the condylar process of the mandible (condylion, Co). Condylar process inclination was defined as the angle of the imaginary line connecting the gonions of both sides and extension of the central axis of the condylar process. A bone area of 5 mm around the fracture site was set as the regions of interest for radio-density measurement.

For radiographic evaluation, panoramic radiographs obtained at postoperative day 1 (POD 1D) and 6-month follow-up (POD 6M) were used. Measurement tools within the PACS (Picture Archive and Communication System) program (INFINITT; INFINITT healthcare, Guro-gu, Seoul, Korea) were used. The distance from the point of the mandibular angle (gonion) to the uppermost point of the mandibular condylar process (condylion) was measured to obtain the ramus height. The angle of the imaginary line connecting the gonions of both sides and the extension of the central axis of the condylar process was measured to obtain the condylar process inclination (Figure 3). To reduce calculation errors, all measurements were repeatedly measured five times by one examiner, and the average of the remaining values, except the maximum and minimum values, was used. In order to minimize the effect of the patient’s facial asymmetry on the results, the ramus height and condylar process inclination were measured for both fractured and non-fractured sides in all panoramic radiographs, and the differences were calculated as follows:

$$\begin{array}{l} \mathrm{Difference}\;\mathrm{in}\;\mathrm{ramus}\;\mathrm{height}\;\\ \;=\mathrm{ramus}\;\mathrm{height}\;\mathrm{of}\;\mathrm{non}\;\mathrm{fracture}\;\mathrm{side}\\ \;-\mathrm{ramus}\;\mathrm{height}\;\mathrm{of}\;\mathrm{fracture}\;\mathrm{side} \end{array}$$

$$\begin{array}{l} \mathrm{Difference}\;\mathrm{in}\;\mathrm{condylar}\;\mathrm{process}\;\mathrm{inclination}\;\\ \;=\mathrm{condylar}\;\mathrm{process}\;\mathrm{incliantion}\;\mathrm{of}\;\mathrm{non}\;\mathrm{fracture}\;\mathrm{side}\;\\ \;-\;\mathrm{condylar}\;\mathrm{process}\;\mathrm{inclination}\;\mathrm{of}\;\mathrm{fracture}\;\mathrm{side} \end{array}$$

If the value of the difference in the ramus height was positive, it meant that the ramus height of the fractured side was shorter than that of the normal side, and if it was negative, it meant that the ramus height of the fractured side was longer than that of the normal side. If the value of the difference in the condylar process inclination was positive, it meant that the condylar process of the fractured side was angulated anteriorly, and if it was negative, it meant that the condylar process of the fractured side was angulated posteriorly.

To evaluate the bone healing of the fracture site, radio-density was measured on panoramic images at POD 1D and POD 6M. The images processing and radio-density measuring were performed with Adobe Photoshop CS5 (Adobe Systems Inc, San Jose, CA, USA) [10]. After matching the contrast, a selection tool in software was used to measure the radiographic densities. The bony area of 5 mm around the fracture site was set as the regions of interest (ROI) for radio-density measurement. The average grayscale values in the ROI ranged from 0 (black) to 255 (white).

In addition, calculated values were analyzed within-group comparisons over time (between POD 1D and POD 6M) and comparison between the two groups (semi-rigid fixation and rigid fixation) within the same time.

### 2.4. Statistical Analysis

Shapiro–Wilk tests were conducted to determine normality in all the statistical analyses of this study. Differences in the ramus height and condylar process inclination over time within each group were analyzed using a Mann–Whitney U test. Differences in the ramus height and condylar process inclination between the two groups within the same time were analyzed using a Wilcoxon signed-rank test. The same nonparametric statistical method was applied to the analysis of radio-density. The difference in operative time between the two groups was analyzed using an independent *t*-test. All statistical analyses were conducted using SPSS ver. 26.0 (IBM Corp., Armonk, NY, USA). The significance level was set at *p* < 0.05.

## 3. Results

Of the 34 patients, 17 were included in the semi-rigid fixation group (14 men and 3 women), and their average age was 37.47 (range 20–77) years. The remaining 17 patients were included in the rigid fixation group (16 men and 1 woman), and their average age was 39.35 (range 22–68) years. Twenty-two patients were treated with the intraoral approach, five patients were treated with the retromandibular approach, and seven patients were treated with the preauricular approach. Thirteen patients had condylar neck fractures, and 21 had condylar process base fractures (Table 1).

Postoperative complications of the procedures are summarized in Table 2. Most patients who had experienced mouth opening limitation after surgery showed improvement with physical training over time, but in the rigid fixation group, there were two patients with limited sliding movement at POD 6M. Two out of five patients who had early occlusal interference were managed by occlusal adjustments and three with prosthetic treatments. In the rigid fixation group, one of the three patients with facial nerve paralysis had temporal branch damage, and two had zygomatic branch damage, and they showed nearly recovered muscular function at POD 6M. None of the patients showed acute infection, condylar head resorption, or malunion. At POD 6M, conspicuous malocclusions, such as posterior open bite, were not observed, and TMJ symptoms, such as arthralgia, were not observed except for opening restrictions.

**Figure 4:**
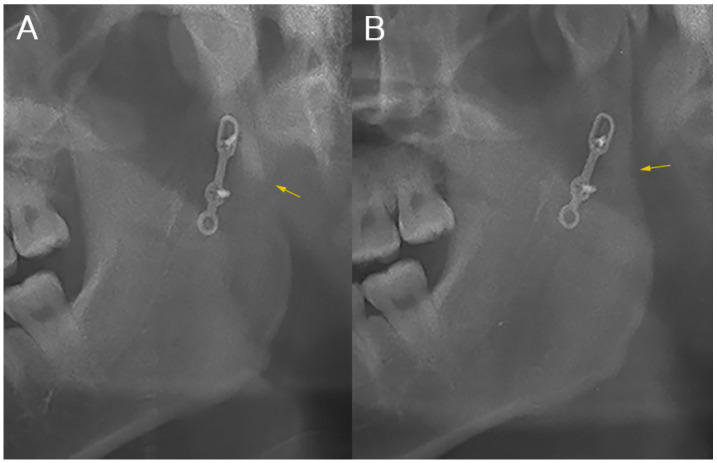
A clinical case in which anterior angulation of the condylar process was normalized at postoperative 6 months. (**A**) In the panoramic radiograph taken at postoperative day 1, posterior ramal bony step remained (arrow) although the condylar reduction was completed; (**B**) In the panoramic radiograph taken at postoperative 6 months, continuous posterior border of condyle can be seen (arrow).

In terms of differences in ramus height, shorter ramus height on the fractured side compared to that on the non-fractured side was observed in all patients. However, there was no statistically significant difference between the two groups, nor was there any difference within each group over time (*p* > 0.05). In terms of differences in condylar process inclination, mild anterior angulation was observed on POD 1D in most patients but also in some well-reduced cases without angulation. When comparing the differences, there was no difference between the two groups, nor was there any difference within each group over time (*p* > 0.05) (Table 3). Surprisingly, in the semi-rigid fixation group, four cases had anterior angulation on POD 1D, which resolved at POD 6M (Figure 4). For radio-density of the fracture site, significantly higher values were observed in the rigid fixation group than in the semi-rigid fixation group at both POD 1D and POD 6M (*p* < 0.05). In both groups, a significant increase in radio-density was observed at POD 6M (*p* < 0.05) (Table 3). In terms of operative time, the semi-rigid fixation group recorded 144 ± 56 min, which was significantly shorter than that of the rigid fixation group (219 ± 114 min, *p* = 0.022).

## 4. Discussion

There is no consensus regarding the optimal treatment for mandibular condylar fractures [11,12,13]. Various studies have reported successful outcomes with non-surgical treatments and dietary restriction in cases of condylar process fractures in adults without displacement as well as in children [14,15,16,17]. Nevertheless, surgical treatment is the gold standard for condylar fractures with displacement in adult patients [18]. Furthermore, if a surgical method is attempted, rigid fixation is considered to be the standard method.

However, it is difficult to place the condylar fragments precisely when rigid fixation is performed because these are usually small, and these can easily get displaced or pulled by the medial pterygoid muscle. It is especially difficult to use the intraoral approach because of its limited visibility. When precise reduction is not achieved, complications, including TMJ pain, restriction of mandibular movement, and malocclusion, may occur. Moreover, resorption of the condylar head may occur when excessive dissection of the intracapsular area is performed for perfect reduction [19]. Therefore, many surgical approaches based on assistance with endoscopes or zigs have been proposed for precise rigid fixation [3,4,20,21,22]. However, successful fixation of condylar process fractures is still a dilemma for surgeons.

Inspired by the intraoral vertical ramus osteotomy (IVRO) concept of orthognathic surgery [23], we attempted to use a sliding plate during the fixation of mandibular condylar fractures. According to the IVRO concept, the articular disc is moved to its original position by the functioning of surrounding neuromuscular complexes without fixation after ramus osteotomy [24]. We expected that the condylar segment would be relocated to its original position after surgery if the mediolateral deviation of the condylar segment could be prevented by the sliding plate. Actually, as expected of the author, as shown in Figure 4, in the case where the anterior angulation of the condylar process was resolved, considering that the measured condylar inclination value changed, it is presumed to be physiological normalization of the condyle head rather than remodeling by bone resorption.

Sliding plates have already been widely studied in the field of mandibular orthognathic surgery [5,6,25,26,27,28,29]. Gursoytrak et al. [25] reported more relapse with the use of sliding plates compared to that with four-hole plates in an animal experiment study. Other clinical studies conducted by Kim et al. and us [26,27] showed no significant differences in the relapse of proximal segments between the sliding plate and four-hole plate groups in orthognathic surgery. Furthermore, studies by Gang et al. [6,28,29] and Roh et al. [6,28,29], who used cone-beam computed tomography (CBCT), and a study by Larson et al. [6,28,29], who used finite element analysis, reported less relapse in terms of condylar position. There is no disagreement in existing studies that sliding plates are effective in stress distribution on the mandibular condyle after orthognathic surgery.

We believe that the application of a sliding plate would have clinical advantages in the open reduction of condylar fractures, similar to that after orthognathic surgery. In this study, when a sliding plate was used for semi-rigid fixation, there were no differences in changes in the ramus height and condylar process inclination compared to those with rigid fixation. Additionally, in semi-rigid fixation, the radio-density of the ROI was observed to be lower than that of rigid fixation, but this was a predictable result because it allowed slight movement of the segment. Rather, it was observed that the radio-density of the ROI significantly increased as time passed, suggesting that the bone healing of the fracture site proceeds well even in semi-rigid fixation. Clinical equivalence of the sliding plate was observed in terms of the expression of various complications when compared with the conventional surgical method. The operative time was shorter in the semi-rigid fixation group than in the rigid fixation group. Furthermore, some cases that were considered to not have achieved a successful reduction in the semi-rigid fixation group showed improvements over time.

On the other hand, the position of the plate and the method of fixation can affect the stress applied to the condylar process. Based on the theory of compression and traction lines in the mandibular condyle proposed by Meyer, placing the plate on the anterior and posterior borders of the lateral side of the condylar process has been a common surgical method for fixation of the condylar process fracture [8]. Since then, many researchers have studied various shapes and positions of plates have been conducted. Murakami et al. displayed that the use of multiple plates rather than the use of a single plate is helpful for stress resistance [30]. Darwich et al. displayed that a trapezoidal plate was best for stress resistance [31], but Liokatis et al. displayed that an alpha or kappa shape rather than a trapezoidal shape was better for stress resistance [32], and de Jesus et al. suggested that a lambda shape is better for stress resistance [33]. In fact, since the author’s study is not a rigid fixation concept, the stress applied to the plate was not considered in the study design. Therefore, the position of the plate was not critically designated. However, efforts were made to position it on the posterior border as much as possible, and a good clinical result was obtained.

Despite these positive findings of using the sliding plate, however, considering the limitation in terms of early recovery of mandibular function, it is difficult to say that the semi-rigid fixation group was better than the rigid fixation group because IMF was applied for two weeks when a sliding plate was used. It was the author’s idea to give the plate space so that the condyle head can move forward and backward and keep the condyle head in the correct position while avoiding a large load during the two-week MMF period. Moreover, the authors were concerned about malunion due to hypermobility of the condylar segment immediately after surgery. Therefore, in order to give the time necessary for callus formation and to minimize the possibility of malunion, IMF was performed for two weeks in the semi-rigid fixation. Despite the shortcomings of the IMF, semi-rigid fixation is not worse than rigid fixation in terms of surgical outcomes. Woo et al. [34] reported that 22 patients with sub-condylar or angle fractures who were treated with semi-rigid fixation and, similar to the procedure used in this study, showed convenience fixation and reliable outcomes.

However, there were some limitations to this study. The first problem is that the data are not free from the influence of the patient’s asymmetry. For supplementation, the non-fracture site was referenced, and the differences of the parameters between the fracture site and non-fracture site were compared, not the simple comparison of the fracture site parameters. However, even the influence of asymmetry caused by the distortion of the panorama image could not be completely overcome. Second, because the data was censored, CBCT could not be used for the analysis. In addition to panorama, it is thought that it would have been a better study if it was possible to investigate the indicators appearing in CBCT or other images. Third, a longer period of analysis over the six months was not performed, and only the clinical course up to six months after surgery was analyzed. Considering that bone-healing ability or loading applied to TMJ varies from person to person, if a longer-term observation is performed, it is thought that the clinical usefulness of the sliding plate can be discussed in more detail.

## 5. Conclusions

This study showed that the reduction of the mandibular condylar fracture using a sliding plate had similar radiological outcomes, fewer clinical complications, and shorter operative time compared to those with a traditional surgical technique, such as rigid fixation. Excluding the shortcomings of the initial limitation of mouth opening, reduction of mandibular condyle fracture using a sliding plate is considered to be an appropriate clinical procedure.

## Figures and Tables

**Table 1:** Classification of patient groups according to the level of the fracture line and approach selection.

	Semi-Rigid Fixation Group (*n* = 17)	Rigid Fixation Group (*n* = 17)
Condylar neck fracture	7	6
Condylar process base fracture	10	11
Intraoral approach	12	10
Retromandibular approach	4	1
Preauricular approach	1	6

**Table 2:** Various complications in the semi-rigid fixation group and rigid fixation group at 6 months postoperatively.

Complications	Semi-Rigid Fixation Group (*n* = 17)	Rigid Fixation Group (*n* = 17)
Mouth-opening limitation	4	6
Early occlusal interference	2	3
Facial nerve damage	0	3
Acute infection	0	0
Condylar head resorption	0	0
Malunion	0	0

**Table 3:** Comparison of differences in ramus height and condylar process inclination between semi-rigid fixation group and rigid fixation group at postoperative day 1 and 6 months postoperatively.

		POD 1D	POD 6M	*p*-Value
Differences in ramus height (mm)	Semi-rigid	4.91 ± 3.79	5.50 ± 4.70	0.705
	Rigid	3.99 ± 2.85	5.35 ± 3.12	0.124
	*p*-Value	0.734	0.786	
Differences in condylar process inclination (degree)	Semi-rigid	6.77 ± 5.06	6.76 ± 8.36	0.723
	Rigid	8.73 ± 10.61	12.30 ± 13.07	0.246
	*p*-Value	1.000	0.708	
Radio-density (Mean grayscale value of ROI)	Semi-rigid	100.5 ± 17.4	102.5 ± 18.4	0.035
	Rigid	120.7 ± 31.4	124.1 ± 28.7	0.044
	*p*-Value	0.041	0.013	

## Data Availability

The data presented in this study are available upon request from the corresponding author.
